# Digital solutions for migrant and refugee health: a framework for analysis and action

**DOI:** 10.1016/j.lanepe.2024.101190

**Published:** 2024-12-26

**Authors:** Stephen A. Matlin, Johanna Hanefeld, Ana Corte-Real, Paulo Rupino da Cunha, Thea de Gruchy, Karima Noorali Manji, Gina Netto, Tiago Nunes, İlke Şanlıer, Amirhossein Takian, Muhammad Hamid Zaman, Luciano Saso

**Affiliations:** a Institute of Global Health Innovation, Imperial College London, London, UK; b Centre for International Health Protection (ZIG), Robert Koch Institute, Nordufer 20, Berlin, 13353, Germany; c University of Coimbra, Clinical and Academic Centre of Coimbra, Faculty of Coimbra, Coimbra, Portugal; d Department of Informatics Engineering, University of Coimbra, CISUC, Coimbra, Portugal; e African Centre for Migration & Society, University of the Witwatersrand, Johannesburg, South Africa; f Charité Center for Global Health (CCGH), Charité Universitätsmedizin Berlin, Germany; g The Institute of Place, Environment and Society, Heriot Watt University, Edinburgh, UK; h Migration and Development Research Center (MIGCU), Çukurova University, Sarıçam/Adana, Turkey; i Department of Global Health & Public Policy, School of Public Health, Tehran University of Medical Sciences (TUMS), Iran; j Departments of Biomedical Engineering and International Health, Center on Forced Displacement, Boston University, Boston, MA, USA; k Faculty of Pharmacy and Medicine, Sapienza University, Rome, Italy

**Keywords:** Migrant and refugee health, Digital health, mHealth, Telehealth, Telemedicine, Ethics, Privacy, Security, Equity

## Abstract

Digital technologies can help support the health of migrants and refugees and facilitate research on their health issues. However, ethical concerns include security and confidentiality of information; informed consent; how to engage migrants in designing, implementing and researching digital tools; inequitable access to mobile devices and the internet; and access to health services for early intervention and follow-up. Digital technical solutions do not necessarily overcome problems that are political, social, or economic. There are major deficits with regard to (1) reliable data on the health needs of migrants and mobile populations and on how they can use digital tools to support their health; (2) evidence on effectiveness of solutions; and (3) a broad framework to guide future work. This article provides a wide socio-technical perspective, as a framework for analysis and developing coherent agendas across global-to-local spaces, with particular attention to the European region.

## Introduction

An increasing array of health applications is being found for digital technologies, including in communication, information storage and retrieval, diagnosis, booking of medical appointments, prescribing and referrals, research, and the management of health services and systems.^1^^,^^2^ This trend was considerably boosted by the COVID-19 pandemic,^3^^,^^4^ as well as by recent advances in applications of artificial intelligence (AI) in healthcare.^5^ Compared to traditional face to face health services, potential benefits to refugees and migrants, including those on the move, encompass increased access to health information, services, and professionals and to their own health records, as well as faster and more accurate diagnosis and more timely and effective treatment that is more patient-centred, patient-empowering, and aligned with integrated systems of care.^6^^,^^7^ For migrants, refugees and other people moving (Panel 1), there are additional potential benefits for individuals and groups who may have very limited access to traditional in-person health services, including in Europe,^8^^,^^9^ for enhancing the self-reliance and resilience of refugees,^10^ as emphasised in the Global Compact on Refugees,^11^ and explored in the Expert Meetings on Digital Solutions for Migrant and Refugee Health in 2021.^12^ Compared to non-digital health services, digital health technologies can also enhance health literacy and support individuals who may fear being stigmatised through targeted interventions, for instance, women who are experiencing Post-Traumatic Stress Disorder (PTSD).^13^

Panel 1 **Migrants, refugees, and other people on the move.**

In this article, ‘**migrant**’ refers to those who have crossed an international border, although we are aware that some of the issues raised here may also apply to **internal migrants** and/or **internally displaced persons**.^14^

**Migrants** include people moving with or without documentation such as personal identity papers and documents confirming rights to visit or reside in the place in which they are currently located.

A **refugee** was defined in the 1967 Protocol of the 1951 Refugee Convention^15^ as a person who, “owing to a well-founded fear of persecution for reasons of race, religion, nationality, membership of a particular social group or political opinions, is outside the country of his nationality and is unable or, owing to such fear, is unwilling to avail himself of the protection of that country”. The 1984 Cartagena Declaration extended this to include persons who flee their country “because their lives, security or freedom have been threatened by generalised violence, foreign aggression, internal conflicts, massive violations of human rights or other circumstances which have seriously disturbed public order”.^16^ Refugees are afforded special protection and entitlements by international agreements.^17^

An **asylum-seeker** is a person who seeks safety from persecution or serious harm in a country other than his or her own and awaits a decision on the application for refugee status under relevant international and national instruments. A person who is denied asylum must leave the country where they have applied and may be expelled.^18^

A **displaced person** is one who has been forced or obliged to flee or to leave her/his home or place of habitual residence, in particular as a result of or in order to avoid the effects of armed conflict, situations of generalised violence, violations of human rights or natural or human-made disasters.^19^

Migrants, refugees, and others on the move such as displaced persons may have poorer health outcomes than the general populations in countries of transit and destination, including in Europe.^8^^,^^20^, ^21^, ^22^, ^23^, ^24^ Discriminatory policies that limit or deny access to health services are embedded in global, regional, and national instruments and are compounded by discriminatory practices and attitudes encountered within services and by fear of detention or expulsion resulting from accessing services.^25^, ^26^, ^27^ There is growing interest in adapting digital tools to assist migrants, refugees, and others on the move such as displaced persons.^28^^,^^29^ However, digital tools can also exacerbate existing vulnerabilities experienced by migrants and refugees due to increasingly tighter border controls across Europe, a phenomenon which has led to the region being referred to by analysts as ‘Fortress Europe’ and to the use of dangerous routes, often resulting in fatalities.^30^ These populations are often rendered vulnerable as a result of xenophobia and discrimination and, in some cases, cross-national systems, such as the Common European Asylum System, and hostile policy landscapes that limit their access to services, including timely healthcare.^31^^,^^32^ It is worth highlighting that digital tools should not be viewed as a panacea for countering inhumane treatment institutionalised in the European border regimes^33^ and in rules causing marginalisation or exclusion from health services, either nationally or regionally, which have been described^34^^,^^35^ as ‘necropolitics’. While it has been argued that digital health could reduce inequality and increase universal health coverage,^36^ in practice migrants, refugees, and those seeking asylum are generally excluded from the “leave no-one behind” principle proclaimed in Agenda 2030 and the goal of “universal” health coverage^37^^,^^38^ and encounter many hurdles to taking advantage of digital solutions as discussed in this article.

Furthermore, in considering the use of digital technologies to improve access to services, it is important to be cognisant of the gendered digital divide in access to these technologies, in addition to their cost, and the use of these digital technologies by States for surveillance and migration control. Reliance on digital technologies to reach these populations may, therefore, further exclude the most vulnerable and expose individuals and communities to the risks associated with increased surveillance, including detention, deportation and discrimination.^39^

Ethical issues and questions regarding the use of digital tools in migrant and refugee health are discussed in Panel 2. Practical concerns have also been highlighted, including issues related to confidentiality, privacy, security, misappropriation and misuse of personal data,^40^^,^^41^ and limitations to access for patients who lack the necessary means, hardware, connectivity, language, culture or skills to use digital tools in the way that they are being imagined.^10^^,^^42^^,^^43^ Moreover, the incorporation of skewed data sets into digital applications may lead to distortions in their use and to perpetuation of existing biases and inequalities.^44^ The actual benefits and disadvantages associated with each digital application consequently depend, among other factors, on where, how and by whom it is designed and used. More fundamentally, the development of these tools should be undertaken as part of the process of decolonising the digital rights field through more collaborative approaches, as is currently being undertaken by European Digital Rights, a network defending rights and freedoms online.^45^ In practical terms, the development of privacy-enhancing technologies which are compliant with the General Data Protection Regulation (GDPR) and a human rights approach can play an important role in safeguarding the rights of migrants and refugees, including those whose residential status is uncertain and who are at risk of deportation.^46^ Understanding the background factors relating to access and use of mobile communications and the internet in general^47^ is therefore crucial to assessing the balance of potential health benefits and harms for each individual, including for migrants and refugees and others who are on the move.

Panel 2 **Ethical concerns and considerations.**

**Background**

The growing opportunities for accessing health information and services afforded by digital interventions need to be considered in the light of the multiple vulnerabilities that may apply to migrants and refugees in different circumstances. For people involved in setting policy, regulating practice, and in offering, operating or using digital approaches, it is therefore vital to take account of ethical concerns, above all operating on the ethical nonmaleficence principle of ‘do no harm’,^48^ set against the normative background of the UN's Universal Declaration of Human Rights^49^ and Sustainable Development Goals (SDGs)^50^ and relevant regional instruments such as the European Convention on Human Rights and its subsequent protocols.^51^

Onarheim et al.^52^ have set out the importance and value of an ethical approach to migration health policy, practice and research, with benefits that include (1) highlighting the inherent normative questions and trade-offs at stake in migration health; (2) assisting decision makers in deciding what is the ethically justifiable thing to do; (3) ensuring that migrants' interests are considered by using ethical frameworks and technical guidance to set normative and practical standards for decision makers facing ethical questions; and (4) responding to the need for greater transparency and accountability in decision making, as well as meaningful participation of migrant groups. Onarheim et al. list a range of ethical issues that need to be considered when dealing with migration health in general, raising questions for health workers, policy makers, data managers and researchers, as well as for international migrants themselves. They also point to the availability of methods to identify ethical issues, frameworks for systematising information and suggesting ethically acceptable solutions, and guidance on procedural concerns and legitimate decision-making processes.

In this article, we focus on the dimension of ethical issues relating in particular to the adoption of digital approaches for migrant and refugee health. Focussing on data protection in migrant and refugee health,^53^ the Migration Data Portal of the International Organization for Migration (IOM), discusses ethical concerns over confidentiality, privacy, security, and misappropriation and misuse of personal data. Providing technical guidance on the collection and integration of data on refugee and migrant health,^54^ the WHO Regional Office for Europe stresses the urgent need for integration of migration health data into every national health information system in order to support the inclusion of refugees and migrants, which became apparent in the COVID-19 pandemic. Bozorgmehr et al.,^55^ while calling for such data to be collected systematically, highlight the importance of attention to safeguarding privacy while combining data from multiple sources, ensuring survey methods take account of the groups' diversity, and the need to engage migrants and refugees in decisions about their own health data. UNHCR cites digital risks, including from online censorship to cyber threats, data protection risks, disinformation and privacy harms, which demand increased attention and action as ‘connectivity as aid’ is mainstreamed as an essential form of humanitarian assistance.^56^

Particular attention has focused on the recent expansion in the use of big data and AI, and it has been suggested that AI provides a test case for rights and that use of AI in decision-making raises ethical questions of fairness and due process.^57^ Floridi and Cowls^58^ have synthesised ethical concerns regarding uses of AI into an overarching framework of five core principles, relating to beneficence, non-maleficence, autonomy, justice, and explicability. Guillen and Teodoro^59^ stress the need to embed AI ethical principles into the design, development, and deployment stages of AI predictive tools for migration management. Taki et al.^60^ argue that research on novel healthcare technologies aiming to benefit forcibly displaced persons such as refugees, who are at an increased risk of physical and mental health conditions, can be conducted under an ethical framework. They observe that, in the areas of omics and digital technology, attention is required to access and connectivity barriers, privacy concerns, guarantee of anonymity and inclusion. To improve the accessibility of clinical research on novel technologies, they emphasise to value of community-based participatory research.

**Questions to consider**

To assist in ensuring a broad coverage of ethical issues in the design and operation of digital approaches to migrant and refugee health, we have assembled a list of questions that should be considered. These cut across the concerns identified above, related to areas that include privacy, confidentiality, security, inclusivity, misappropriation and misuse of personal data, respect for rights and freedom of choice. The list of questions has been grouped under three headings and framed in a way that points to practical approaches for adoption.

**Data protection**

• Does use of the app or platform expose the participant/patient to potential surveillance risks? Is it possible to create a firewall between the app or platform and immigration and police services?
• What data is being collected by the app and/or platform in the background? Who is collecting it? And what will be done with it now and what might be done with it in future?
• If there is a data breach, could the information collected expose the participant/patient to identification and/or risk? Are there ways to minimise this?
• What identifying information will be collected? Is it possible to collect less?
• How and where will the data be stored and de-identified/anonymized? Who will have access to it? When will the data be destroyed?
• What safeguards are in place to ensure that data shared via the app or platform is not misappropriated or misused by those who currently have access to it and those who may have access in the future if the app or platform is sold?
• Is the app or platform sufficiently secure so that if another person uses or steals the participant/patient's device, they will not have access to the information? Is it easy for the participant/patient to delete the app or their interaction with the service or research from their device?

**Informed consent**

• How does the target population use mobile devices and apps? What are the security concerns regarding the use of these? Are they aware of other risks associated with sharing personal information through apps and on mobile devices?
• Has due attention been paid to ensuring that processes of informed consent have considered the most commonly used languages of potential users?
• Can the consent process outline—in a clear and concise manner—the purpose of the app or intervention and what data is being collected, including in the background, and what risks are associated with this approach, specifically in relation to data protection?
• Are there ways through which participants/patients can request that their data be deleted or modified?
• Will changes to the app or platforms ownership and data storage approaches be clearly communicated to participants/patients until the data is deleted?
• Is information about the complaints process included? Is this information available in the languages most commonly used locally?

**Exclusion and inclusion criteria**

• Does the use of the app or platform exclude certain groups of people, specifically due to their gender, age, documentation status or financial situation? How will this affect the results of the research or the impact of the service?
• What additional barriers to access care or participate in research may the use of an app, platform and/or mobile device create? Are there other approaches being simultaneously employed to ensure that those who cannot or do not want to use the app, platform or device are not excluded from the research or unable to access services?
• Are there ways to circumvent potential participants/patients' initial discomfort or scepticism regarding the app or platform, for example, through an in-person initiation?
• In research involving digital solutions for migrant health, does the research design, conduct and analysis and reporting involve meaningful migrant participation at every possible stage and have the research processes and tools been assessed to ensure that benefit to the migrants and protection of their privacy and security are prioritised?

The Minoritised Ethnic People's Code of Practice for Equitable Digital Services (ME-CoP) has been provided to guide decision making about the purpose of digital services, their design, delivery, and use of people's data, including but not limited to, race and ethnicity information. Through seven principles,^46^ it recommends how the design of digital services can help safeguard against some of the inequities minoritised ethnic people experience in access, outcomes, and their experiences of services. The principles have been co-created with minoritised ethnic community members as experts by experience, and also stakeholders and representatives from across healthcare, third sector, social housing, design consultancy, public sector, local authorities, UK regulators, and the data science community.

Evidently, there is a need for policies, strategies, and tailored tools to maximise the benefits while safeguarding the individuals and for more research to understand the advantages and risks. Research related to these issues may itself be facilitated by the use of digital tools, but again with need to consider both users and subjects in relation to benefits and risks and having regard to both ethical and practical factors.

As noted by Mancini et al., the realm of digital migration studies has been fragmented and lacking an analytical focus,^61^ while the importance of a combined socio-technical perspective has been emphasised.^62^ The variations in circumstances that are significant for migrant and refugee individuals and groups include their locations, with important factors including laws and practices that relate to the opportunities and constraints they experience in accessing health and digital connectivity. In the European region, countries in general have high levels of health service coverage and of digital connectivity nationally,^63^^,^^64^ as well as inter-country reciprocal agreements providing cross-border health coverage and supporting digital roaming. Migrants and refugees may nevertheless find their access to these services severely constrained,^65^ especially when having unclear resident status.

This Perspective article draws on the expertise of a number of researchers with extensive personal experience of working with migrants and refugees (including some from the Global South and, in some cases, having experienced migration themselves), combined with an extensive search of the literature. The origins of this article in a World Health Summit Expert Meeting, its development and the methods employed are set out in Panel 3.

Panel 3 **Background and methods.**

**Background**

The World Health Summit (WHS) Academic Alliance (formerly known as the M8 Alliance of Academic Health Centers, Universities and National Academies) has organised World Health Summit annual meetings since 2009.^66^ These meetings bring together policy-makers and people working in international and non-governmental agencies and academia, with the goals of agenda-setting for global health improvement and development of science-based solutions to global health challenges. Since 2015, the Alliance has put migrant and refugee health on the agenda of the annual Summit meetings and since 2017, led by Sapienza University of Rome, has held Expert Meetings, either in Rome or online, attended by participants from around the world, on migrant and refugee health.^67^ An online meeting in 2021, co-organised by Sapienza University of Rome and the London School of Hygiene and Tropical Medicine,^68^^,^^69^ discussed the subject of digital solutions for migrant and refugee health. Subsequently, the presenters in this meeting agreed to collaborate to develop a broad-ranging overview of digital solutions for migrant and refugee health, combining knowledge from their own on-going work in the field with the growing body of literature on digital aspects of health and placing this in the evolving technological, social and political contexts of particular relevance in the European region.

**Literature search strategy and selection criteria**

Co-authors contributed to the identification of themes and topics, through the discussions in the original Expert meeting and subsequent exchanges as they contributed text to the draughting, with incorporation of results from their own ongoing work and from the emerging literature. To complement and extend the literature presented by the co-authors which informed the initiation of this article, a search was implemented across Google, Google Scholar, PubMed, Scopus, ScienceDirect, and open-access documents from pertinent organisations, including the International Organisation for Migration, World Health Organization (WHO), and European Commission. Combinations of terms relating to digital technology in general and specific forms (e.g., eHealth, mHealth, telemedicine) were combined with ‘health’ and the terms migrant, refugee, asylum seeker or displaced person. Additionally, to refine the scope and consider factors such as regional and ethical dimensions, keywords such as “Europe” “European” and “ethics” were incorporated. The final reference list was curated based on the relevance and quality of the studies regarding the topics within the extensive scope of the review, as well as priority for papers published in the last five years and papers concerning the European region.

The article summarises key opportunities and challenges in the use of digital technologies for the health of migrants and refugees. These opportunities include wide accessibility of digital tools through mobile phones among people on the move, the continued increase in digital literacy, the possibility of developing context appropriate solutions (e.g., apps) with smaller investments (compared to non-digital solutions) and accessibility and protection of medical records that otherwise can be lost in the chaos of migration, to name a few. There are important ethical and technical challenges that are also discussed in this paper. This introductory section has contextualised the research against a global background, focussing in particular on the European context. We intend to use a socio-technical perspective to offer a broad, multi-dimensional framework both for analysis and for developing recommendations for action across global-to-local spaces. Key terms and ethical guidelines have been outlined. Next, the article explores digital tools for health in the general population followed by a discussion which pays detailed attention to key aspects relating to the use of digital tools for health for migrants and refugees. The article then considers the use of digital tools in research in two respects: use by migrants and refugees of digital applications for health and utility of digital applications to study migrant and refugee health. This is followed by a discussion of issues related to equity and inclusion. Finally, we draw together the framing of our analysis and recommendations for analysing and developing digital tools for migrants and refugees.

## Digital tools for health: general population

In the WHO Global Strategy on Digital Health,^70^ the term digital health encompasses eHealth (information and communication technologies for health),^71^ mHealth (medical and public health practice supported by mobile devices),^72^ and other uses of digital technologies for health such as the Internet of Things (IoT), advanced computing, big data analytics, artificial intelligence (AI), robotics, telehealth, telemedicine, and personalised medicine. The Global Strategy notes that “digital health should be an integral part of health priorities and benefit people in a way that is ethical, safe, secure, reliable, equitable, and sustainable. It should be developed with principles of transparency, accessibility, scalability, replicability, interoperability, privacy, security, and confidentiality”.

The 2023 State of Digital Health report^73^ shows extremely variable progress towards the goals of the Global Strategy^70^ across WHO regions. The report assesses most countries at the western side of the WHO European region to be at a mature phase (Phase 5) of development in digital health, while some at the eastern side of the region are at a lower phase (Phase 4). The report calls for more action on equity and inclusion, strengthening workforce skills, the development and implementation of architectures and standards that promote interoperability between digital health interventions to support the continuum of care, and more investment in infrastructure and services.

While a wide range of potential benefits of digital tools have been proposed for health providers and patients, there have also been a number of concerns expressed, including about the need for AI ethics and governance to keep pace with the rapid technical advances being observed.^74^^,^^75^ Cummins and Schuller^76^ pointed to challenges they considered crucial to overcome to ensure that digital health systems meet the guiding principle of being “for all anywhere and at any time”. Five groups of challenges were highlighted: (1) societal (regulatory and legislation factors, perceived lack of commercial sector accountability, complexity of the multinational nature of the digital health market operating within a multitude of different health systems, varying levels of digital and health literacy in the general population, especially in the elderly, data ownership, and other ethical concerns); (2) ethical (role of consumer technology companies in collecting, storing, and analysing health data); (3) increasingly connected health solutions (patient safety, security and privacy concerns in transferring data from the point of collection, such as IoT devices, to remote servers and the degree to which patients and research subjects understand how their data is being processed and by whom); (4) role of AI (safety, explainability, and fairness, lack of standards for verification and validation); and (5) potential of genomics (uses of and sharing of information derived from genetic profiling).

In addition, we must recognise the xenophobia and continued poor treatment of refugees and migrants at the European borders^77^^,^^78^ as well as within countries.^25^ Digital health solutions—no matter how precise or accurate in identifying the cause or the symptoms, or providing a guideline for care—would be impotent in an environment that degrades humans and disregards their basic human rights. There have been several studies to date, demonstrating how, at the borders of the EU, migrants and refugees are treated with a disregard to their humanity, and the power dynamic between border guards and migrants results in ill-treatment and exploitation.^79^, ^80^, ^81^ Digital interventions—and cross border partnership between countries—therefore must not operate in a vacuum where technical interventions are disconnected from ground realities of violation of human rights and must include equity in digital rights as a central feature.^82^

## Digital tools for health: migrants and refugees

### Framing health in broader contexts

**Fig. 1:**
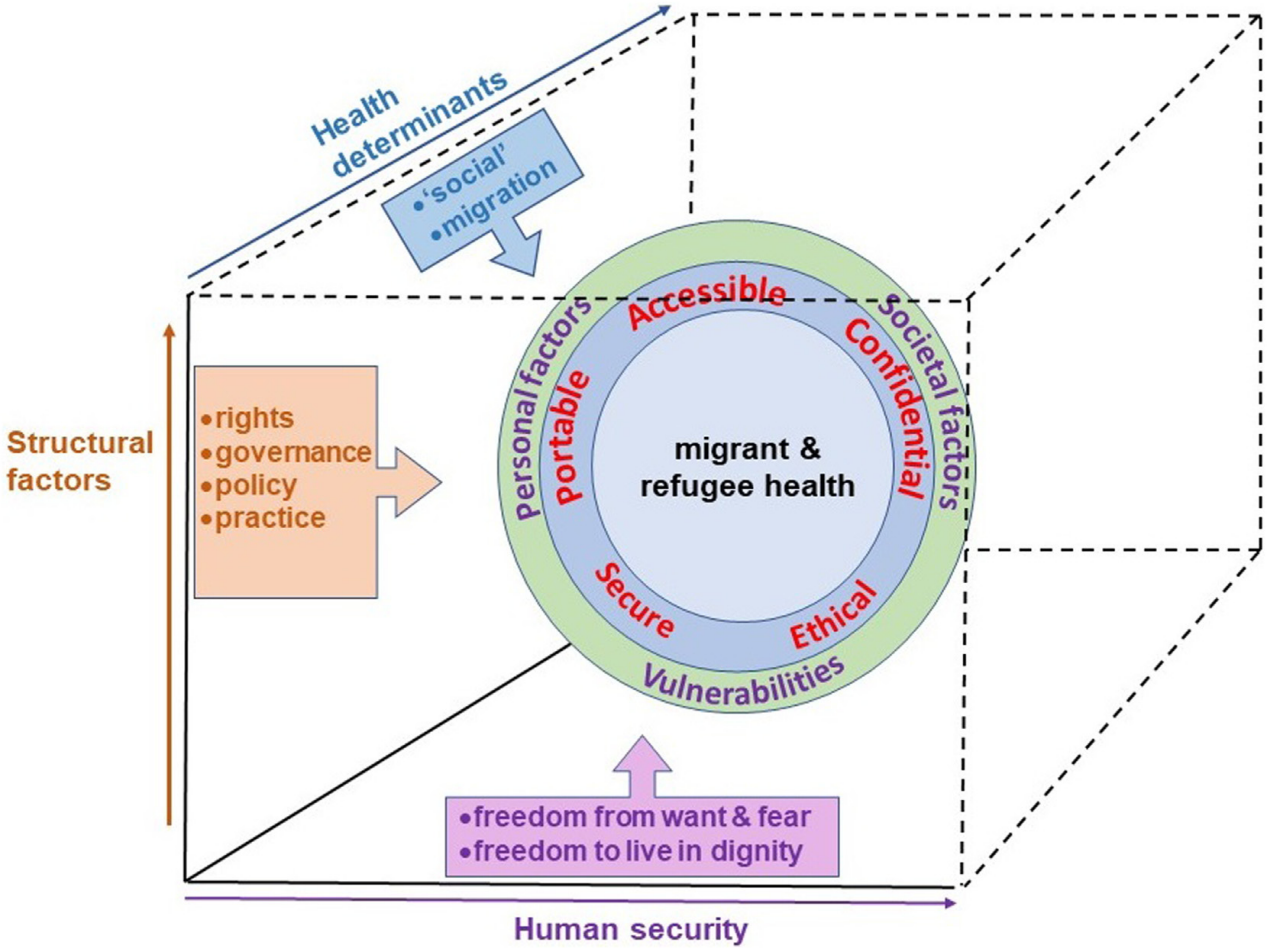
Dimensions and factors relevant to the use of digital tools for migrant and refugee health.

For migrants and refugees, the totality of events, experiences and circumstances leading to their current situation may include diverse stresses and traumas along their migration pathway.^20^^,^^22^ Multiple, complementary perspectives are therefore necessary. Interwoven with the potential benefits, risks and challenges of digital health in relation to the general population categorised by Cummins and Schuller,^76^ there are additional human factors that need to be considered with regard to the use of digital tools to support the health and health-seeking behaviours of migrants and refugees, including personal and societal factors and vulnerabilities. A broad framework for analysis was developed earlier to examine gaps in migrant and refugee health (Fig. 1).^83^ The constellation of dimensions and multiple factors relevant to the use of digital tools for migrant and refugee health is illustrated in Fig. 1.

This model represents the health of migrants and refugees as being at the centre of concentric spheres of influence, most immediately involving human factors (personal and social factors and vulnerabilities). A further sphere of influence (see WHO Global Strategy^70^) involves technological factors, with important requirements including the need for digital solutions to be accessible, confidential, ethical, secure, and portable. This space in which migrant and refugee health resides is subject to many further influences, which the model combines into three complementary perspectives considered to be of particular significance, grouped in three dimensions as follows:

• Structural factors:
  Deficits have been observed in all aspects of structural factors that are embedded in states and systems, including rights, governance, policies, practices, equity and social justice, that affect the health of migrants and refugees^27^^,^^83^ and have been highlighted in discussions of the WHO Global Action Plan.^84^, ^85^, ^86^ Migrants and refugees are often ‘exceptionalised’ by governments,^87^ despite commitments to rights for all and to ‘leave no-one behind’.^88^, ^89^, ^90^, ^91^ The concept of ‘structural vulnerability’ has been presented in relation to the challenges of clinical care and healthcare advocacy for migrants, aiding consideration of how specific social, economic and political hierarchies and policies produce and pattern poor health.^92^ There has been criticism of interoperability, biometrics, and identity management in the EU, including concerns for the lack of equity built into in these structural arrangements which become entangled with security politics^93^, ^94^, ^95^, ^96^ and demand participation by groups concerned with digital rights.^97^
• Health determinants:
  The Dahlgren-Whitehead model of health determinants incorporates a range of biological, behavioural, sociocultural, economic, and ecological. Factors that are contained in the core categories, or pillars, of nutrition, lifestyle, environment, and genetics, with medical care as a fifth pillar to support them.^98^ Further extension of this model has come from understanding the importance of individual experience of events as a contributory factor to good health or ill-health.^99^ In this context, the experience of migration itself is recognised as a significant determinant of health, with contributary factors coming from the individual's experiences, before, during and after the migration process.^24^^,^^100^ As an example, a systematic review found that restrictive entry and integration policies are linked to poor migrant health outcomes in high-income countries and it was noted that efforts to improve the health of migrants would benefit from adopting a Health in All Policies perspective.^101^
• Human security:
  Defined^102^ as “freedom from want and fear and freedom to live in dignity”, the concept of human security incorporates threats to security in health, food, environmental, economic, personal, community, and political domains. It has been adopted by the UN as the basis for an integrated approach for the realisation of Agenda 2030.^103^ Further, digital inclusion and digital technologies have also been recognised as determinants of health.^104^^,^^105^

In the context of digital solutions for the health of migrants and refugees, the involvement of these components of Fig. 1 is exemplified in the sections below. In this multi-dimensional, multi-factor perspective, the health of an individual migrant or refugee emerges from dynamic, complex, interacting ecosystems^106^ that alter with spatial and temporal factors. Analysis and formulation of policy and strategy need to incorporate system-level understanding and systems and spatial thinking if they are to avoid the pitfalls of siloed approaches.^107^, ^108^, ^109^

### Mobile technology and connectivity: availability, access, affordability, and rights

The capacity of those who are in the process of migrating or experiencing displacement to access and use mobile devices and the internet can be crucial factors contributing to their survival and wellbeing.^110^ The multifunctional mobile phone enables many opportunities, including maintaining contact with family and friends, obtaining information related to security threats, facilitating travel, access to food, water, shelter, and work, providing channels for remittances, and seeking health assistance.^28^^,^^62^^,^^111^, ^112^, ^113^ For many refugees, internet and mobile connectivity have a level of importance similar to basic needs such as water, food, and energy,^114^, ^115^, ^116^ in this sense bridging the dimensions of health determinants, human security and structural factors such as human rights (Fig. 1).

Migrants moving through regular channels and able to have a residential address and financial resources will often see the establishment of a mobile phone account at their new location as one of their immediate priorities. For those who have migrated irregularly, arranging mobile access may be one of their greatest concerns, whether or not health issues are an immediate problem.^117^ In some circumstances, possession of a mobile phone is so important that it may be the subject of barter, extortion, blackmail or physical conflict.^61^ The UN High Commissioner for Refugees (UNHCR) asserts^118^ that displaced populations and communities that host them have the right, and the choice, to be part of a connected society, with access to technology that enables them to build better futures.

For some migrants and refugees, including in Europe, barriers to their mobile phone usage may include confiscation (whether legal or not) by authorities.^119^^,^^120^ In addition, studies have noted that internet infrastructure can be unreliable, incapable of handling a high volume or deliberately denied, thereby creating a substantial challenge for communities on the move to access information, health and other essential services that rely on robust communication.^121^ Other constraints include difficulties accessing devices that have the capacity to operate the applications and features needed and receive security updates, lack of a power supply, costs and documentation requirements associated with SIM cards,^56^ access to charging points,^122^ and lack of the required documentation to register for a mobile money account^111^ and an underlying income problem often linked to restrictions on rights to move and work.^123^ Effort and resources are needed to ensure that mobile ecosystems mature equitably and inclusively.^111^^,^^114^^,^^124^

Reception conditions in Europe often lack adequate access to the internet and WiFi, which becomes especially critical during health emergencies, such as the COVID-19 pandemic. During such times, access—or rather the lack of access—to the internet has emerged as a significant barrier for refugees and asylum seekers in seeking health information and maintaining communication with individuals and organisations. As services and information rapidly shifted to the digital realm, the digital divide exacerbated existing vulnerabilities. For instance, refugees faced challenges in accessing essential health information, social services, and even basic daily needs, such as ordering food or sanitary supplies while under quarantine. This digital gap has been highlighted in various reports^125^, ^126^, ^127^ from multiple countries. In Germany, for example, research has shown that the lack of digital infrastructure in reception centres left many refugees unable to access crucial health information, particularly during the pandemic.^128^ Similarly, in Greece, a study found that the limited availability of internet and WiFi in refugee camps severely restricted the ability of refugees to access online health services and essential daily needs.^129^ Moreover, in Italy, the lack of affordable mobile data plans for refugees has been cited as a significant barrier to accessing online platforms for health information and social services.^130^

The failure to provide adequate digital infrastructure not only hampers access to health information but also impedes the fulfilment of basic social and humanitarian needs.^121^ For instance, during quarantine periods, the inability to order food or access sanitary products online left many refugees in precarious situations. Addressing these infrastructural deficiencies is therefore crucial, as they serve as barriers to essential health information and the fulfilment of basic needs, ultimately impacting the well-being and integration of refugees.

Adopting a patient-centric approach, in which a patient is the owner of their data and may allow hospitals and health professionals access to their data,^131^ implies that the patient has access linguistically to the data owned and requires, in the case of migrants, the potential for multilingual operation of the data.^132^ At the present time, automated machine translation—especially in specialised technical areas like health and medicine—is not sufficiently advanced to be reliable without human participation in verification and editing.^133^^,^^134^

Digital inclusion of refugees has been framed by humanitarian agencies as a fundamental human right and essential tool to promote access to education, health care, social connections, income, and skills development.^135^ Lack of access by migrants to Internet and WiFi, including in reception conditions and during health emergencies, represents a loss of digital rights and barrier to accessing essential services.^121^ Recent critical literature highlights significant concerns regarding digital rights and the healthcare of migrants and refugees, particularly around issues of privacy, consent, and data security on one hand and rights of access on the other. For instance, the increasing use of digital technologies in humanitarian contexts can lead to unintended consequences, such as the surveillance and profiling of vulnerable populations, which can exacerbate existing inequalities and discrimination.^135^, ^136^, ^137^ Latonero^138^ has discussed the ethical implications of digital data collection in refugee populations, emphasising the need for strict data protection measures to prevent misuse by both state and non-state actors. Gillespie et al.^139^ explored the tension between the potential benefits of digital health tools and the risks associated with digital exclusion, surveillance, and the erosion of privacy rights for migrants and refugees. Digital exclusion prevents access to health information and services has been reported for migrants seeking asylum, for example in the UK.^121^^,^^140^ These critical perspectives highlight the need for improvements in policy and practice, underscoring the importance of adopting a rights-based approach^141^ to the development and deployment of digital health solutions, ensuring that the rights and dignity of migrants and refugees are upheld. They point to the need to make digital approaches for the health of migrants and refugees ‘safe and beneficial by design and in operation’.

### Opportunities and risks

For the migrant, opportunity and vulnerability to risk are associated with digital and mobile technologies in ways that vary with time and place along the migration pathway,^62^^,^^142^, ^143^, ^144^ constituting an infrastructure^145^ for ‘digital passages’. These have been described^138^ as “sociotechnical spaces of flows in which refugees, smugglers, governments, and corporations interact with each other and with new technologies.”

A scoping review^146^ of digital health interventions for ethnic or cultural minority and migrant populations, published in 2023, found that about two thirds were developed for communities in the USA and the remainder aimed at communities spread across Europe, Asia, Africa, and Australia. The review noted that addressing this unequal distribution in the future is important as population diversity and heterogeneity are significant factors. Moreover, the studies included focused showed a general tendency to prioritise the development of digital health interventions for people with a settled legal status over those with a precarious or unclear immigration status. The technologies that were most widely used included mhealth interventions (35%), websites and informational videos (23%), text messages (14%), and telehealth (14%). Most applications were aimed at illness self-management, followed by consultations and prevention, with the main health issues addressed being mental health and wellbeing (23%), pregnancy and postpartum (17%), and overall lifestyle habits (15%).

In a commentary on using digital health technologies as a possible solution for improving accessibility to essential healthcare services or mitigating the health consequences in migrants when conventional service approaches are unavailable, Hou et al.^147^ observed that there remains an unrealised opportunity for investments in digital health to address migrants’ health needs. They noted that, while digitalised health offers the hope of providing cost-effective, mobile health services that can help to overcome structural barriers to achieving the highest possible health level in migrants, a number of changes are necessary. These relate to reinforcing production and synthesis of evidence through research, fostering collaborations across national and local multi-stakeholders, and empowering migrants.

However, in the case of migrants, refugees, and asylum-seekers the growing opportunities for digital interventions to benefit health must be weighed against the potential disadvantages and risks, which can vary substantially with the locations and circumstances of each individual and connect with the recognition of migration as a determinant of health, with personal security and human rights (Fig. 1). An area of particular concern that poses a substantial threat that may apply to large numbers of individuals relates to European border regimes, which have become increasingly dehumanising in recent years and failed to respect human rights in their treatment of those seeking refuge and asylum.^148^, ^149^, ^150^ The Council of Europe Commissioner for Human Rights has highlighted four areas for urgent action to end the human rights violations taking place at Europe's borders and that relate to pushbacks involving the summary return of refugees, asylum seekers, and migrants by states without the observance of the necessary human rights safeguards. These areas involve the need for member states to re-focus on the implementation, in good faith, of their human rights obligations, in particular those set out in the European Convention on Human Rights; to enhance transparency of border control activities, in particular through strengthening independent monitoring to prevent and identify violations, as well as bolstering mechanisms to ensure accountability when such violations occur; and to acknowledge pushbacks as a pan-European problem requiring collective action by all member states; and for parliamentarians to mobilise to stand up against pushbacks, including by holding their governments to account and by preventing the adoption of laws or policies that are not human rights compliant.^81^

Both analogue and digital information is used by police and border guards seeking to identify and exclude refugees and asylum-seekers even before their cases have been properly examined. Because of the ease with which it can be obtained, amassed, searched and exchanged, digitalised data is of particular concern.^151^ The possession of a mobile phone, generally regarded as a vital resource for a migrant (see section "Mobile technology and connectivity: availability, access, affordability, and rights") can also increase the person's vulnerability, for example, to surveillance.^152^^,^^153^ A digital infrastructure for movement can easily be leveraged for surveillance and control. Fear of detention and deportation may deter migrants and refugees from seeking health care in a timely manner and this adds significant further risk to their health.^154^^,^^155^ Reports that in some countries, including in Europe, immigration authorities extract mobile phone data in order to identify migrants who may be undocumented or to ascertain whether asylum seekers are “lying” reinforces these fears.^156^, ^157^, ^158^

In a scoping review of studies on the role of mobile phones on refugees' experience, Mancini et al.^61^ reported that mobile phones have sometimes become a form of currency, to be bought and sold, exchanged and bartered, fought over and gifted. Obtaining new SIM cards can present risks of exploitation, exposure or being tracked.^159^ Digital technology, especially social media networks, also provide mechanisms to circulate evidence of the suffering experienced by refugees on social media platforms—serving as a ‘digital witnesses’ which may help to address human rights abuses, but may also be a risk to the lives of those holding or transmitting the information. Thus, ensuring that individuals can remain invisible when they so wish is imperative.^62^ Positive associations have been observed between technology-enabled social connections and overall well-being, including in the areas of mental health and facilitation of information relating to health and health care,^160^ while negative aspects of the use of digital media include technology-facilitated domestic violence against immigrant and refugee women.

A UNHCR report^56^ identified a number of digital risks, including online censorship, cyber threats, data protection risks, disinformation, and privacy harms. These risks demand increased attention and action while “connectivity-as-aid”^118^ is mainstreamed as an essential form of humanitarian assistance. The report^56^ noted research showing that, despite awareness of the issues, connected refugees often feel powerless to do much about online threats and digital risks to their security and privacy online, while policy environments related to telecommunications access, such as SIM registration requirements, may introduce risks to vulnerable users.

In more than 100 countries, including the Member States of the European Union (EU), the right to privacy or private life enshrined in the Universal Declaration of Human Rights is expressed, among other ways, by the operation of data protection laws that aim to preserve the confidentiality of personal data,^161^ including the GDPR in the EU. However, for migrants and refugees, being able to keep information confidential, to have a private life and to be secure from harm, detention or expulsion may be very difficult in practice.^162^ The difficulties may be further compounded by factors such as gender and age. Mobile phones offer a secure way to connect with family, friends and helpers through encrypted media such as WhatsApp, but the use of the phone may entail risks that the user may be tracked, plans for movement intercepted or phone lists used to identify contacts of interest to criminals or authorities.^61^^,^^135^^,^^163^ The risks, as well as opportunities, have increased in recent years^5^ with the growing use of AI tools by migrants (e.g., employing ChatGPT) and by government authorities (e.g., using AI pattern recognition capacities).

### Digital literacy and the connected migrant

With the growing use of smartphones and social media such as Facebook and WhatsApp around the world, including across most emerging economies,^164^ the important contribution that digital health literacy can make to helping to limit inequalities from expanding has been highlighted.^104^^,^^165^ Among other factors, this requires a digitally literate health workforce,^166^^,^^167^ having competencies^104^ in four areas:

• Functional: the ability to successfully read and write about health using technological devices;
• Communicative: the ability to control, adapt, and collaborate communication about health with others in online social environments;
• Critical: the ability to evaluate the relevance, trustworthiness, and risks of sharing and receiving health-related information through the digital ecosystem (e.g., the Internet); and
• Translational: the ability to apply health-related information from the digital ecosystem (e.g., the Internet) in different contexts.

The importance of digital literacy for the ‘connected migrant’ has also been emphasised.^168^^,^^169^ Refugees in particular settings have been characterised as subject to a digital divide, digital exclusion or being ‘digitally unprepared’.^121^^,^^170^, ^171^, ^172^ Reports also highlight the wide spectrum of capacities and skills in digital literacy observed in practice and the requirement for better alignment between needs and provisions to mitigate concerns that access to technology can potentially exacerbate inequalities within refugee communities, including along the lines of proficiency in the dominant languages used at the local level.^10^^,^^62^^,^^110^^,^^173^, ^174^, ^175^ A 2021 state-of-the-art review^160^ of digital skills in refugee integration, spanning the major resettlement regions (North America, Western Europe, Oceania), highlighted, among other aspects, the systematic overlooking of the perspectives and preferences of refugees in the development of digital apps to assist integration, as well as neglecting variations in, and the need for training in, digital skills to navigate and judge capably the reliability of internet resources.

Digital health literacy is a critical component in addressing health inequalities, particularly as digital tools become increasingly integral to accessing healthcare and health information. However, it is important to recognise that digital health literacy is one of many factors that contribute to health outcomes. Health inequalities are influenced by a complex interplay of determinants, including socioeconomic status, education, cultural barriers, and access to healthcare services. While digital health literacy can empower individuals to better navigate digital health environments and make informed health decisions, it should not be seen as a “super determinant”^165^ that can independently mitigate all health disparities. Rather, it is a valuable piece of a larger puzzle (c.f. Fig. 1) that requires a comprehensive, multi-faceted approach. Ensuring equitable access to digital tools, alongside improving general health literacy, enhancing socioeconomic conditions, and addressing systemic barriers, is crucial to effectively reduce health inequalities.

### Digital identity

Lack of official documents establishing identity can limit a person's access to resources, services and socio-economic participation. It is estimated that there are 680,000 stateless persons currently residing in Europe, often lacking basic documents such as a birth certificate, identity card or passport.^176^ Establishing a digital identity may be an alternative, but it has been argued that the technologies and processes involved in digital identity will not provide easy solutions in the migration and refugee context, while they introduce a new sociotechnical layer that may exacerbate existing biases, discrimination, or power imbalances that are among the structural factors influencing migrant and refugee health (Fig. 1).^177^ Case studies in Italy reported in 2019 observed that migrants exchanged identity data for resources without meaningful consent, while privacy, informed consent, and data protection were compromised throughout the process of migrant and refugee identification. Moreover, there were systemic bureaucratic biases that would likely impede the fair development and integration of digital identity systems. A stronger evidence base and appropriate safeguards were priorities, without which new digital identity systems were likely to amplify risks and harms in the lives of vulnerable and marginalised populations.

Digital identities and biometric data, such as fingerprints and iris scans, are increasingly employed to ensure migrants and refugees can access essential services and claim their rights. While these measures are vital for legal identification and service provision, they pose significant risks, including heightened surveillance, privacy violations, and potential abuses by state and private actors.^178^^,^^179^ Unlike the general population, migrants are often subject to mandatory biometric registration, leading to disproportionate surveillance and control justified under security or administrative needs. Additionally, when private companies manage these systems, there is a risk of commercial profiling and exploitation, further complicating the balance between securing rights and protecting individual privacy. These challenges highlight the need for robust data protection, informed consent, and transparent governance to ensure that identification measures do not compromise the rights and freedoms of vulnerable populations.

The concept of the “smart refugee”, characterised by self-monitoring, agility, entrepreneurship, and resilience,^113^ reflects an individualising rhetoric that aligns with neoliberal ideals of self-sufficiency and marketability. However, this narrative can obscure the broader structural and political contexts that shape refugees' lives, reducing complex social realities to simplistic notions of individual capability and responsibility. Van Dyk and Haubner^180^ critique this framework as symptomatic of a “community capitalism” that, paradoxically, emerges even as neoliberalism faces its own crises and pivots away from extreme individualism towards more collective approaches. Furthermore, Brunnett^181^ argues that the focus on individualised medicine often neglects the social determinants of health and the political power dynamics that influence access to care and health outcomes, particularly for marginalised populations such as refugees. Integrating these critical perspectives highlights the need to move beyond reductionist narratives of the “smart refugee” and towards a more comprehensive understanding of how systemic inequalities and power relations impact health and well-being.

### Apps for health

There has been a growing number and range of uses of smartphones and apps for health applications, both by health professionals and patients^182^^,^^183^ and the field has been extensively covered in systematic reviews.^184^, ^185^, ^186^, ^187^, ^188^, ^189^, ^190^, ^191^ In this section we focus on uses for the health of migrants and refugees. These have broadly fallen into two, sometimes overlapping, areas: (1) provision of health information and service gateways for those not able to access other local services^192^ and (2) provision of assistance to facilitate access, communication, and integration.^193^

One advantage of digital health interventions is that they can be deployed rapidly in conditions where physical access may be limited, as was demonstrated during the COVID-19 pandemic in the provision of an mHealth application to increase access to preventive maternal and child health services for Syrian refugees in Turkey.^194^ However, digital communication, in itself, can be a paradoxical factor in relation to health, as was seen during the COVID-19 pandemic, either amplifying existing inequalities in access to health care for many migrants (as was seen in a UK study,^195^ attributed to a lack of digital literacy and access to technology, compounded by language barriers) or reducing psychological distress but increasing health-related risk perception (as was seen in a study^196^ of refugees in Italy). Furthermore, a lack of empathy perceived by migrants or refugees in interactions with healthcare providers can lead to misunderstandings, especially in cases with limited language skills and/or health literacy, which may be exacerbated by remote interactions or use of AI tools.^197^^,^^198^

Many migrants and refugees experience psychological problems, including as a result of stresses and traumas before, during and after migration, feelings of loneliness and isolation from family and friends, anxiety about their own and their family's situation and fear of being detained or deported.^199^^,^^200^ Communication difficulties often act as barriers for migrants and refugees seeking help with mental health issues.^201^ A systematic rapid review^202^ highlighted the strong need for language support (with remote language facilities, including multilingual electronic systems, being options to consider) and development of cultural competence in mental health services. A systematic review of electronic tools for bridging language gaps concluded that there was need for rigorous evaluation of their acceptability, efficacy, and actual use.^203^

A 2023 systematic review by Abtahi et al.^13^ of how digital interventions are implemented to address the mental health and well-being of international migrants found few studies that involved delivery of the intervention rather than use of technology in the research process. The reports included in the review showed evidence of benefit in interventions for depression, efforts to increase mental health literacy, targeted health promotion, the aiding of social connections, and the alleviation of Post-Traumatic Stress Disorder (PTSD). As in other literature,^204^ the review by Abtahi et al. noted that the stigma associated with mental health problems can be a barrier to seeking treatment and that digital interventions may allow a person to receive mental health services with more privacy, without the knowledge of their family, friends, and community. However, the results of the two RCT studies examining stigma included in the review were mixed, with Kiropoulos et al.^205^ finding lower personal stigma scores in the Internet-based intervention group for depression than the control group, while the study by Nickerson et al.^206^ of an online stigma reduction intervention specifically designed for refugees (‘Tell Your Story’) reported no significant effects for self-stigma for PTSD. A 2024 integrative literature review^207^ of digital mental health interventions for the mental health care of refugees and asylum seekers reported a few more recent studies that suggested benefit in overcoming shame, but overall concluded that further research is needed to confirm effectiveness.

A study of Arabic-language digital interventions for depression in German routine health care found them acceptable, but adoption of the digital interventions remains a challenge and requires facilitation and tailoring.^208^ Examination of ‘Step-by-Step’, a digital psychological intervention for refugees in Egypt, Germany, and Sweden, identified diverse factors that influenced scalability of the intervention in each country.^209^ The complexity of interrelated factors and actors involved pointed to the need for multi-stakeholder collaboration, including the involvement of end-users, being essential for integrating novel e-mental health interventions for refugees into routine services. Rafftree^210^ has explored how digital approaches and interventions could be incorporated safely and feasibly into the different layers of mental health and psychosocial support services for displaced and stateless adolescents.

With the market proliferating for mental health apps that are designed to help refugees manage symptoms of PTSD and other mental health issues, Abdelrahman^211^ has noted that these apps are part of a larger endeavour to create the ‘smart’ refugee who is self-monitoring, agile, entrepreneurial and resilient in the face of adversity, However, she cautioned that these apps are harvesting, storing and selling information on refugee trauma and experience of loss, grief and suffering as marketable commodities. As such, ensuring that firewalls are put in place to protect data shared through these apps from being shared with immigration or law enforcement agencies, as well as from the private sector, is imperative if access to healthcare is predicated on use of these apps.^212^ Examining digital technology to address chronic illnesses in moving populations, Osae-Larbi^213^ identified the need for the strategic development and adoption of ‘realistically smart’ phones. These would be affordable and designed to have, in addition to the basic features of a mobile phone, capacity for wireless internet connection; a built-in or affixed sensor for measuring multiple vital health information; and a core set of approved medical and health apps preinstalled.

### Health records and uses of blockchain

Migrants and refugees may face difficulties in obtaining appropriate and timely diagnosis and treatment due to absence of health records, pointing to the need for creating electronic health records.^214^ As indicated by a systematic review,^215^ electronic health records that are portable and that can be accessed from any location by those authorised may be efficient and effective tools for registering, monitoring and improving the health of migrants and refugees, with potential to address some of the challenges that they face in accessing health care. The importance of user-centred design in the creation of such tools has been emphasised.^216^

Blockchain (BC) technology, which involves tamper-evident and tamper-resistant digital ledgers,^217^ is increasingly used in fields where data security and confidentiality are important.^218^ It is generally claimed to be robustly secure with regard to cybersecurity, data privacy and the IoT.^219^, ^220^, ^221^, ^222^ Furthermore, BC is claimed to enhance users' control over the data generated using web applications, since it prevents enterprises providing network applications from privately storing user interaction data.^223^

However, concerns have been raised about the security and privacy-related challenges derived from BC's complexity, scalability, lack of standardisation, and diversity of protocols,^224^, ^225^, ^226^ and about whether use of BC is compliant with EU data protection law.^227^ Examining the feasibility of new humanitarian applications for blockchain, Connolly et al.^228^ surveyed current theoretical and practical work on how BC can be used to help protect the human rights of migrants and refugees, primarily through creation of digital identities. In a critical examination of cases, they found BC can be useful in empowering vulnerable individuals, but there are also potential human rights risks, such as the infringement of privacy and discrimination, and it is recommended that adequate safeguards should be in place to ensure that BC initiatives meet their true purposes of protecting the most vulnerable groups. Dimitropoulos^229^ critically examined the adoption of BC by international organizations, including those in the humanitarian sector, raising questions about the legitimacy of use *per se* of digital distributed ledgers, and the risk that, while giving someone a digital identity, a very robust, hard-to-change record is created that collects everyone's data. Highlighting the importance of accountability of public power, Dimitropoulos called for a new social contract for blockchain to ensure that its use by international organisations and all public institutions supports the goal that no one is left behind in the digital era, both in terms of means of subsistence, as well as basic political rights. Self-sovereign identity (SSI)—user-controlled, decentralised forms of digital identification—is closely linked with the distributed ledger technology and is proposed as a tool to empower marginalised groups, including refugees. Some advocates claim that SSI removes the need for powerful, centralised state and corporate structures by giving individuals control and ownership of their identity information,^230^ which is a vital asset in contexts of migration circumstances where an individual's identity documents become lost or inaccessible. Cheesman^231^ has challenged these claims, arguing that the reality of competing factors related to four issues (the neutrality of the technology, the capacities of refugees, global governance and the nation state, and new economic models for digital identity) act to destabilise SSI's potential as a tool of refugee empowerment rather than state or corporate control.

Applications of BC in health^232^, ^233^, ^234^, ^235^, ^236^, ^237^, ^238^, ^239^, ^240^ include preserving and exchanging patient data through hospitals, diagnostic laboratories, pharmacy firms, and physicians; contributing to identifying severe and dangerous medical mistakes; improving the performance, security, and transparency of sharing medical data in the health care system; enhancing the analysis of medical records; and responding to the COVID-19 pandemic. A number of features claimed for BC technology are of particular relevance to migrants and refugees.^228^^,^^241^, ^242^, ^243^, ^244^ These include the core requirements (Fig. 1) for digital health to take account of personal and societal factors and vulnerabilities while being accessible, confidential, ethical, secure, and portable. To date, the main uses of blockchain in support of migrant and refugee health have been in addressing lack of personal identification and unavailability of health records.^242^^,^^243^^,^^245^^,^^246^

In the context of this evolving picture of the use of BC to provide security, confidentiality, and privacy in personal data records, Panel 4 presents a discussion of the employment of BC for health records and the potential benefits and challenges for their application to the health of migrants and refugees, with a focus on the European Region.

Panel 4 **Blockchain insights: transforming health records in Europe.**

The European Health Data Space emphasises the efficiency of health systems during a crisis, individual ownership of data, access, and the global impact of data reuse.^247^ Its objective is to overcome shortcomings in communication, interoperability, security, privacy, and data quality^247^ caused by European countries' fragmentation of standards and specifications.^5^^,^^242^ By 2025, the Member States, except for Denmark and Romania, will be required to implement electronic health records (EHR) in a standardised format (EHRxF),^248^^,^^249^ including information on medicines and health data (Myhealth@EU).^250^ Similarly, in 2022 the OECD also addressed the implementation of EHRs through cohesive regulation and governance, referencing Denmark and Finland.^251^ Globally, EHRs must promote functional, structural, and semantic interoperability, mitigating data heterogeneity.^242^^,^^252^^,^^253^ Additionally, quality, record certification and access to data are contemplated and mandatory in the Europe standards.^247^

In centralised systems, the transfer of information between two entities is overseen by a third-party organisation, which validates and completes the transaction.^254^ Implementing European data protection regulation standards in closed domains, protected with firewalls and intrusion detection systems, is possible through centralised management.^254^ However, the applicability of this model is questionable, given the growing volume of data.^255^ Additionally, there are interoperability challenges due to the incompatibility of the information systems, particularly in the cases of Germany, Spain, and Italy.^253^ In a decentralised network, several entities communicate and coordinate with each other to maintain a coherent system for the users.^254^ The design challenge resides in managing the replicas, ensuring consistency of all the copies of data distributed across several parties, and dealing with security, transparency, fault tolerance and management, scalability, and load balancing.^256^

An example of a decentralised network that can be used to manage health records is blockchain (BC) technology.^257^ This is a distributed ledger technology used in domains where trust is a fundamental concern.^242^^,^^244^^,^^256^, ^257^, ^258^ A block is defined as a set of data or a collection of records. Once a block is full, another block is created and added to the network as part of a chain through a mining or validation process. The information is replicated through a distributed network of peers and, once written, cannot be modified. New records can be added after peer validation using a consensus algorithm.^257^ BC chronologically records information in a tamper-resistant data record, transacted or broadcasted across a network of peers or users.^258^^,^^259^ This allows any legitimate user or node to participate in the network and read and write in its ledger. A consortium BC creates a decentralised environment, a collaborative ecosystem, where no third party controls the transaction and the data.^257^ The data is shared and available to all nodes, which makes the system transparent. The immutability and transparency of the blocks constituting the ledger enable tamper-proofness and traceability of the data source.^257^ From an individual entity perspective, security is achieved by assigning a unique identity associated with their account and restricting access to personal records exclusively to the user.^242^^,^^260^ The veracity of data held also depends in part on the protection it receives from external attackers, and preventing unauthorised access is contingent upon the network's privacy features.^261^ This protection is closely linked to the network's privacy model.^242^^,^^256^^,^^261^ While applications of blockchain technology are growing rapidly, there is are significant skills gaps,^262^, ^263^, ^264^ including in relation to the need to develop capacity of health workers and patients to access and use health records appropriately, skills of professionals to critically assess applications in areas such as migrant and refugee health, and lack of experts, investment, and infrastructure.

BC is associated with EHR in a distributed workflow that is consented, authorised, and regulated. This safeguards medico-legal issues and the implementation requirements of a health data management network.^243^^,^^244^ BC can be employed in inter-organisational workflows as a disruptive technology with the potential to enhance the accessibility and reliability of the information, facilitate data sharing, and provide users with the ability to input and review information in real-time, particularly in the context of identity, health, law, or academic records.^244^ In 2021, the Expert Meetings on Digital Solutions for Migrant and Refugee Health explored the potential of blockchain technology.^265^^,^^266^ The potential of BC to address the issues of migrants and refugees was introduced in Estonia^260^^,^^267^ and subsequently it has been considered for restructuring cross-border flows, scaling diasporas, and globalising identity data.^242^, ^243^, ^244^^,^^256^^,^^258^, ^259^, ^260^^,^^265^^,^^266^ A secure and verifiable blockchain-based or self-sovereign identity integrated into certified platforms would provide migrants and refugees access to cross-transactional records.^242^^,^^267^^,^^268^ Estonia provides an example of the introduction of BC technology and its applications across a range of e-government functions. Since 2007, Estonia has been at the vanguard of rethinking the security of personal data, utilising BC in a hybrid public-private digital signature model^269^ and making available a public key infrastructure card which gives access to EHR on the Guardtime platform.^267^ This has helped reduce the economic and social vulnerability of the migrant population in Estonia by including them in e-Residency which provides access to Estonian e-services and can be used in online environments for personal identification and digital signing,^270^ affording e-Residents digital identity^271^ and access to secure financial transactions.

Due to its considerable importance, BC has been the subject of conceptual and empirical investigation, including ad hoc implementations by companies and other organisations in recent years.^243^^,^^256^ Regarding interoperability solutions, blockchain networks have been identified as a key component in the design of database architecture for research platform pilot projects, particularly from a bottom-up perspective, focussing on the homeless population.^243^

Besides technological progress, the digitalisation of healthcare is also shaped by regulation, particularly the GDPR^244^ in the EU. Following the 2019 pandemic crisis, it was necessary to analyse the regulatory framework.^272^ In 2021, the European Council proposed the creation of a cross-sectoral legislative framework on the collection, access, storage, use, and re-use of healthcare data, addressing specific challenges and compliance with European values, with an emphasis on maintaining a complete history of medical data, the development of personalised medicine, and the quality of healthcare.^247^ The transparency of civil and medical data is a highly sensitive issue in the different jurisdictional scenarios within Europe.^55^ The potential for conflict between the GDPR and the immutability of BC can be mitigated by a number of means, including the review of existing laws and regulations, the implementation of compliance mechanisms, the utilisation of hybrid network architectures and governance frameworks for privacy assessments, and the pursuit of a balance between the rights of the individual and the impact on the community.^244^ This can be achieved by anonymisation or by following ethical guidelines and standards (e.g., transparent policies on secure and responsible storage) in accordance with *leges artis*, theoretical concepts, and basic moral principles adapted to the new challenges of the digital age. Integrating compliance mechanisms, auditing processes, and privacy-enhancing technologies in the context of BC use can be a pivotal strategy, capitalising on privacy-enhancing technologies such as encryption or pseudonymisation. The combination of BC with off-chain storage to store or delete sensitive data can be employed to store hashes of that data, which serve as “fingerprints” attesting to the integrity of the data off-chain. It would be prudent to consider the implementation of governance frameworks with a view to ensuring compliance with data protection legislation and the mitigation of potential privacy risks.^244^^,^^256^

### Artificial intelligence

Applications of AI in the health field are increasing^273^ and present both opportunities and risks for migrants and refugees. The potential benefits for the health of migrants and refugees, include the ability to identify and proactively target migrant groups which may be particularly vulnerable to certain illnesses. For instance, African and Caribbean men are at higher risk of contracting prostate cancer and may benefit from access to screening tests, thus supporting expanded access to services. Such efforts need to be considered alongside the risks of use of AI to identify, trace, and monitor individuals, as well as the perpetuation of existing biases and discriminatory processes that become embedded in the algorithms and data sets incorporated in the AI.^274^

## Research

Research relating to digital solutions for migrant and refugee health has two main aspects. One relates to investigations about the use of digital applications by migrants and refugees for their own health needs. The other concerns investigations of the value of the use of digital applications by researchers as a means to gather data on migrant and refugee health. There is growing interest in such research, including in cross-cutting opportunities afforded by advancing technologies and in the methodological and ethical challenges particular to research in this field.^275^, ^276^, ^277^ A cross-cutting theme reflected in the literature is the need for inclusion of migrants, refugees and displaced persons—those who may otherwise be invisible to or be harmed by traditional research tools and methods—in the whole research process, based on clear ethical criteria.^60^

While quantitative studies offer important data on migration and refugee health, incorporating qualitative research methods rooted in humanities and social sciences, including ethnographic studies, is crucial for capturing the complex and sensitive nature of refugee experiences.^97^^,^^143^^,^^146^ Ethnographic research, such as that described in *Fresh Fruit, Broken Bodies*,^278^ provides a deep understanding of the social, cultural, and economic factors that shape migrant lives and health outcomes. This type of research allows for a more nuanced exploration of the lived experiences of refugees and migrants, often revealing systemic inequalities and barriers to accessing healthcare that quantitative methods alone might overlook.

Moreover, qualitative studies like those conducted by Willen^279^ on unauthorised migrants' experiences of healthcare in Tel Aviv and Khosravi^280^ on undocumented migrants in Sweden further underscore the importance of ethnographic approaches in highlighting the everyday realities and struggles of migrant populations. These studies illustrate how qualitative methods can capture the voices and perspectives of migrants, providing a richer context for understanding their health needs and challenges.^281^

In addition to ethnographic studies, participatory research approaches are needed in refugee and migration-related research, aiming not only to enhance the validity of the research by incorporating the perspectives of the participants but also to contribute to empowerment of the participants and their communities and providing them with a platform to share their stories. Such approaches actively involve refugees and migrants in the research process, ensuring that the findings are more relevant and reflective of their lived experiences. Recent examples of participatory research include storytelling and arts-based activities, which have been used to explore migrants’ experiences of integration and well-being and support adjustment^282^ and the Photovoice initiative with Syrian refugees in Lebanon which allowed participants to document their daily lives and health challenges through photography, with self-reports from some participants highlighting changes in posttraumatic stress, anxiety, and somatic symptoms over the course of programming.^283^

With the aim of promoting health equity for marginalised groups, a continuum of approaches to participatory health research (PHR) have been developed, with a list of more than 25 ranging in type from academic-driven research to equitable shared decision making between academic and community partners.^284^ This plethora of approaches has raised questions including what constitutes PHR, how to evaluate the impact and added value of PHR, how to adapt PHR to specific areas of application, and what clarifications are important in ethical questions in PHR.^285^ Rustage et al.^286^ highlight that two fundamental principles of participatory research that underpin the ability for stakeholders to effectively co-operate and share power are those of inclusivity and democracy, encapsulated in the questions, “has the research included the individuals the research would otherwise be about, and have these individuals, during their inclusion, had influence or power over research decisions on par with the research professionals?”

In the first systematic review to robustly measure the application of participatory approaches and principles to health intervention research for migrants, Rustage et al. note that there are varied interpretations as to how to apply participatory approaches. For example, they found instances of active participation of migrants, proxy participation and indirect participation in the 28 studies selected for review (only a handful of which involved a digital tool as an integral component, such as a video or social media message), and while all the studies involved non-academic stakeholders in at least one stage of the research, only two showed evidence of active participation of migrants across all research stages. The authors conclude that participatory approaches to developing health interventions for migrants are insufficiently applied and reported and that the application of approaches does not fully embody core principles of participatory research, particularly relating to providing decision-making power to individuals ultimately affected by the research. They recommend that those wishing to engage in participatory research must consider the approach they take and critically analyse whether it is sufficient to achieve high-quality participation, not just high-quality research. They also emphasise the crucial need for introduction of guidelines for reporting of participatory research methods, as a prerequisite to explore the overall impact of participatory research, which currently remains inadequately understood. The International Collaboration for Participatory Health Research working group on migration has presented a position statement^287^ addressing opportunities, challenges and ways forward in relation to migrant health. They note that, for each research context, it is essential to gauge the ‘optimal’ level and type of participation that is most likely to leverage migrants' empowerment.

A framework for refugee and migrant health research in the WHO European Region has been provided by the WHO Regional Office for Europe.^288^ The framework discusses three interrelated dynamics in research practice, namely research prioritisation, study samples and research design, offering recommendations to consider for each. It emphasises the value of involving refugees and migrants in research and research agendas and the need to develop an ecosystem that will support and sustain participatory, interdisciplinary, transdisciplinary, and inter-sectoral projects.

Participation of migrants and refugees themselves is a core requirement for block chain. As a cross-cutting digital tool, blockchain affords opportunity for a range of applications that can benefit migrants and refugees including in healthcare, financial matters and oversight of programmes such as for asylum and migrant integration and the exposure of human rights abuses. As Ardittis observed, “Although not a panacea, blockchain could offer major cost-efficiency, transparency and accountability benefits for future migration and asylum programmes”.^289^ Given the sensitivity of data security and confidentiality, it is important that the risks attendant on adoption of BC are carefully considered in every case, including for data collection and management in both service programmes and research, with primacy always being given to the interests of the subjects through their participation.

### Research on use by migrants and refugees of digital applications for health

Data on the health status and needs of migrants and refugees at population levels is generally sparse and more is required. The World Bank's Handbook^290^ offers guidance to support Member States in the collection, tabulation, analysis, dissemination and use of migration data, to contribute directly to monitoring the implementation of the SDGs. The Handbook highlights that the 2030 Agenda to “leave no one behind” has significant implications for data collection, since policy makers need to identify and address all under-served groups, so that data must be disaggregated by migratory status, including in the health field. Potential innovative, big data sources of migration data are indicated, including mobile phones, online tools and platforms such as social media or online payment services, and digital sensors and metres such as satellite imagery. The Handbook advocates a multisectoral approach to health, with policy responses addressing the underlying social determinants of migrants' health (as illustrated in Fig. 1) and the barriers that prevent migrants from accessing quality health care services.

Social media platforms and other digital interventions can enhance uptake of health prevention and promotion services among migrants and refugees, such as health information services, vaccinations, and health check-ups, as demonstrated by evidence relating to the COVID-19 pandemic period.^291^, ^292^, ^293^, ^294^, ^295^, ^296^ This is an area where research could help advance understanding of the factors involved and provide more evidence on effectiveness of such interventions.

A National Academy of Medicine report in 2019 emphasised that, while technology has the potential to support individuals across the mental healthcare continuum and is already being extensively used, it raises a complex web of ethical dilemmas.^297^ While the report did not specifically refer to migrants and refugees, its focus on ethical factors in the areas of privacy, rights, trust, and transparency, make the findings of particular relevance to these groups. To address the concerns identified, the report called for a number of actions from decision-makers and other stakeholders, including creating a governance structure to support the broad and ethical use of new technology in mental healthcare, developing regulation grounded in human rights law, embedding responsible practice into new technology designs and adopting a “test and learn” approach in implementing technology-led mental healthcare services in ways that allow continual assessment and improvement and that flag unintended consequences quickly.

A rapid review^298^ of digital health applications in mental health care for immigrants and refugees found that satisfaction and positive attitudes were generally reported by participants. However, there was generally poor implementation and reporting of the ethical standards of the digital health application studied. A systematic review^299^ of the literature on smartphone-delivered mental health care interventions for refugees also noted that participants were mostly satisfied, but levels of provision fell far short of the needs. Among other findings, the review identified room for improvement in the efficacy and effectiveness of smartphone-delivered interventions and in data safety, as well as the need for more knowledge about effective treatment elements and the barriers that hamper wider use in refugee populations. To achieve high acceptance and utilisation among refugees, the review recommended developing culturally and contextually adapted interventions with high attractiveness and trustworthiness, as well as intervention approaches that are as diverse as possible to address the heterogeneity of the target population.

Technology-based interventions allowing support and treatment for mental health to be provided to refugees offer a number of advantages, including the opportunity to deliver help rapidly and remotely to difficult locations, and the possibility to use digital innovation to deliver interventions while reducing reliance on specialists.^300^ However, to be successful, the need was also emphasised for digital interventions to be in line with the capacity and motivations of the target audience, focussing attention on the vital importance of user-centred design in developing digital interventions.

### Research on utility of digital applications to study migrant and refugee health

Innovations in digital technologies offer increasing opportunities to conduct research with migrants and refugees, including research related to their health and health-seeking behaviour.^301^, ^302^, ^303^ Digital devices and apps may provide opportunities to overcome some of the myriad methodological challenges of primary data gathering in challenging settings for example, where research participants are on the move or may be put at risk by exposure through research methods that force them to be visible, where there are cultural and linguistic differences, and where participants may be retraumatized by face-to-face research methods. Missing or incomplete sampling frames are also a challenge that may be assisted by digital devices and apps, but much more attention is needed to understand the benefits and limitations of these approaches.^304^^,^^305^

The growing use by migrants and refugees of smartphones and social media^306^^,^^307^ has encouraged exploration of the use of social media platforms as digital research tools.^308^^,^^309^ A scoping review examined published health research that uses WhatsApp as a data collection tool that offers opportunities to maintain contact and participation across time and place and that can interface with online platforms that allow for the automatic administration of surveys.^310^ The papers reviewed largely used WhatsApp to send hyperlinks to online surveys, or to deliver and evaluate either an intervention designed for healthcare users or a communication programme for healthcare providers. Notable findings included a lack of attention in publications to the experiences of research participants while interacting with the WhatsApp interface, to the impact of the studies, or to research ethics, including protecting participants’ privacy. Recommendations for researchers included the need to systematically and clearly document and discuss their use of the application when presenting their research, pay greater attention to data privacy and security through selecting and recording only necessary information and encrypting the recorded data so that it is only available to the researchers, removing identifying information, saving the data on secure servers, and making greater efforts to ensure that participants understand the terms of the research and are provided with information, relating to the specifics of the research project and how they can seek and access support should it be required.

There has been success with research that has used WhatsApp to understand the intersections between migration, mobility, health and gender in South Africa^311^ and conduct the first survey with migrants and refugees from sexual and gender minorities in South Africa.^312^ This research points to the potential of WhatsApp as a research tool, whilst highlighting the need for researchers to proactively engage with the ethical implications of this work as it takes place outside of a well-established framework for ethical research.

However, there have been increasing concerns expressed about data protection and the lack of security of global social media platforms, including WhatsApp.^313^, ^314^, ^315^ A complaint that WhatsApp Ireland Limited was forcing users to accept its processing of their data if they wished to continue using the service was upheld.^316^ WhatsApp's compulsory privacy policy is not uniform globally, but incorporates its right to collect a wide range of personal identity and location data.^317^ An independent review into the use of WhatsApp and other instant messaging applications within the police service in the UK^318^ identified risks of non-compliance with data protection legislation and of data breaches and disclosure as being areas of real concern and recommended the development of guidance on when the app could be used in police work. In the light of these wide-ranging concerns, researchers wishing to use social media platforms need to ensure they have up-to-date information on security and privacy risks when contemplating the use of the platforms in their studies. It should be required that this information is presented as part of an application for approval of the research.

## Equity and inclusion

Many factors, including gender, age, ethnicity, cultural background, migration status and language skills, as well as digital literacy, influence opportunities and capacities to take advantage of digital tools and services, including those related to health.^319^^,^^320^ While overall use of mobile phones and the internet in Europe is high, there is a significant difference between usage rates for men and women, with a gender gap of 6–8% being reported. Usage rates are much lower in many low- and middle-income countries and gender gaps higher,^321^ and these gaps may contribute to lower overall and gender-related gaps in digital access and use in migrant populations in Europe.^322^ Ethnicity is also a significant factor in the health inequalities seen in and between host and migrant populations. Such inequalities may be exacerbated or potentially alleviated by digital health care, as has been discussed in the UK.^323^^,^^324^ Such factors present a compelling argument for taking an intersectional approach to examining access and use of digitalised health services, which considers not only the impact of social identities, but also geography. The absence of attention to many areas of diversity, including aspects such as disability and LGBQTI, in the digital space has been highlighted,^325^, ^326^, ^327^ and movements initiated towards decolonising digital rights.^45^^,^^328^^,^^329^ Verovic^330^ has stressed the importance of a multidimensional perspective on diversity in the migration context, both in terms of moving beyond the ethnic group as either the unit of analysis or sole object of study and by appreciating the coalescence of factors which condition people's lives. An intersectional approach illuminates health disparities and the underlying structures that create and maintain disparities. The use of intersectionality theory^331^^,^^332^ to consider the ways in which experiences of racism interact with sexism and other systems of oppression in individuals' experiences of health sciences is still new, for instance in the UK.^333^ However the use of the theory in health sciences is growing^97^^,^^334^^,^^335^ and is currently being extended to investigation into the use of digitalised health services in the UK.^323^ Sabik^336^ has provided an intersectionality toolbox as a resource for teaching and applying an intersectional lens in public health.

Inclusion is also an important factor in the process of developing apps and digital services to support the health of migrants and refugees. A 2023 Scoping Review looked at digital health interventions developed for ethnic or cultural minority and migrant populations, the health problems they address, their effectiveness at the individual level and the degree of target population participation during development. About half did not involve the target population in development and only a minority involved them consistently, while the increased involvement of the target population in the development of digital health tools led to a greater acceptance of their use.^146^

Bozorgmehr et al.^55^ have commented that coverage of migrant and refugee data is incomplete and of insufficient quality in European health information systems. To create healthcare policies and practices that are truly inclusive of migrants and refugees, they have proposed four approaches, involving (1) strategies that ensure that data is collected, analysed and disseminated systematically; (2) methods to safeguard privacy while combining data from multiple sources; (3) enabling survey methods that take account of the groups’ diversity; and (4) engaging migrants and refugees in decisions about their own health data. In support of the last approach Quyoum and Wong^44^ have argued that the use of co-design methods can strengthen capacity amongst racialised communities and stakeholders to articulate where inequities are occurring, increase understanding of how to counter harm, and co-create solutions to ensure that digital services are equitable and responsible by design. The involvement of refugees in the development of applications through transdisciplinary research is also illustrated through two studies building on evidence generated from qualitative research and participatory approaches in the same project from a social science and human computer interaction approach, respectively.^12^^,^^45^ Elements of this collaboration which may be regarded as good practice include early consultation with a refugee-led organisation in determining the focus of the study; financing the organisation to engage with the research; pre-workshop meetings with participants to build trust and rapport; the inclusion of trusted community workers in the facilitation of the workshops; exercising gender and linguistic sensitivity throughout the process; feeding back the results to participants, to validate the research findings and identify ways forward; and participation in the planning of dissemination activities. The authors argue that these collaborative processes not only enabled them to build up trust and rapport with key individuals within the refugee community, but also contributed to the development of more ethically developed and effective digital tools.^337^ In another example of good practice, the Hera app, developed as a social enterprise model to ensure sustainability, bridges the gap between Syrian women refugee women and the care they and their children need, by allowing the user to carry their medical records with them on their smartphone. The app sends reminders when immunisations or check-ups are needed, and also contains relevant emergency information, such as the location of the nearest hospital.^338^

## Framing analysis and action

As noted in the UN's roadmap for digital cooperation,^339^ “digital technology does not exist in a vacuum—it has many sociotechnical facets that have enormous potential for positive change, but can also reinforce and magnify existing fault lines and worsen economic and other inequalities”. Digital tools are not a panacea. The technical and equity problems generally associated with digital technology, including access, identity, security, confidentiality of information, risks of abuse, exploitation and fraud, also apply to and may be magnified for migrants and refugees. They may change the balance of opportunities and risks each individual faces in seeking solutions to their daily challenges, including in health. Digital tools provide technical solutions: they do not necessarily overcome problems that are political, social, or economic and that originate beyond the technical sphere (Fig. 1), although they may be able to help. For example, they may facilitate access to information, but do not replace the need for health workers to acquire cultural competence as an essential tool^340^ in their engagement with migrants and refugees.

Hou et al.^147^ have observed that “addressing the intersecting challenges of migration and healthcare service accessibility demands a holistic approach that calls for both feasible solutions and policy supporting the implementation of solutions”. While much attention has been focused on how digital approaches can potentially assist with challenges of accessibility to health information and treatment,^341^^,^^342^ as discussed in this article, relatively little has been given to the implicit assumption that improving accessibility when conventional service approaches are unavailable will lead to improved clinical outcomes. Hou et al.^147^ also observed that migrating affects healthcare service accessibility, continuity, quality, and equity through the diverse and complex needs of migrants generated depending on geographic conditions, pre-existing health issues, exposure to new health risks through migration and settlement, food security, mental health, livelihood, and other non-health factors which result in health vulnerabilities, including but not limited to gender, age, ethnicity, and religion. All of these factors mediate the extent to which accessibility translates into improved clinical outcomes, with digital health approaches able to facilitate, cost effectively, better recognition of health problems and potential solutions by both patients and health providers. But they do not to overcome structural and social factors in the provision and uptake of treatment. Reviews of the use of digital approaches for migrant and refugee health highlight the lack of evidence on clinical outcomes, including lack of data on the magnitude of inequalities in infection risk, disease outcomes, consequences of pandemic measures or explanations of underlying mechanisms.^343^

Available evidence on the effectiveness of digital approaches in producing improved clinical outcomes is mixed. For example, one of the areas most studied has been in mental health. A randomised controlled trial of a WHO-guided digital health intervention for depression in Syrian refugees in Lebanon was effective in reducing depression in displaced people,^344^ and a scoping review on the impact of digital technology on the well-being of older immigrants and refugees concluded that use of digital technology benefited the well-being and quality of life of older immigrants and refugees, including helping them cope with migration-induced stress.^320^ However, a systematic review of technology-based mental health interventions in minimising mental health symptoms among immigrants, asylum seekers or refugees found scant evidence that the use of digital interventions, such as mobile-based therapies, video conferencing, and digital platforms, was associated with a statistically significant reduction in depressive and anxious symptoms.^345^ Implications include that health service providers and researchers need to gather more evidence on the effectiveness of digital interventions and acquire greater training themselves in the use of digital technologies for health. Implications for policy makers and service planners include the need to provide channels and mechanisms through which migrants can acquire greater understanding of the opportunities and risks associated with digital solutions and enhance their skills in using digital approaches.

Set in the context of the SDGs, the UN Road Map for Digital Cooperation has the goal of ensuring that every person has safe and affordable access to the Internet by 2030, including meaningful use of digitally enabled services, taking a people-centred approach that leaves no one behind.^339^^,^^346^ The Road Map^339^ stresses the vital importance of digital inclusion, requiring better metrics and greater attention to the situation of people on the move, including migrants and other vulnerable communities who are often absent from digital cooperation discussions and face additional challenges in achieving connectivity.

In September 2024, the UN Summit of the Future included a digital technology track leading to a Global Digital Compact, billed as the first comprehensive global framework for digital cooperation and AI governance, which was issued as an Annex to the Summit's Pact for the Future, along with a Declaration on Future Generations.^347^ The Global Digital Compact espouses numerous principles and commitments of direct relevance to migrants and refugees, including closing all digital divides between and within countries, recognising the need to identify and mitigate risks of new technologies, having the goal of an inclusive, open, sustainable, fair, safe and secure digital future “for all”, fostering an inclusive, open, safe and secure digital space that respects, protects and promotes human rights, promoting digital accessibility for all and supporting linguistic and cultural diversity in the digital space, committing to connect all persons to the internet, including the needs of people in vulnerable situations and those in underserved, rural and remote areas in the development and implementation of national and local digital connectivity strategies, and expanding inclusion in and benefits from the digital economy “for all”. However, the Compact makes only one direct reference to migrants, in paragraph 13 committing to “develop and undertake national digital inclusion surveys with data disaggregated by income, sex, age, race, ethnicity, migration status, disability and geographical location and other characteristics relevant in national contexts, to identify learning gaps and inform priorities in specific contexts”. This focus on identifying migration status rather than dealing with any other aspect of migration may not be understood as congruent with the proclamation of rights and benefits for all permeating the rest of the text. It is now important that organisations concerned with migrants and refugees work for the interpretation and implementation of the Compact in ways that support and benefit their rights, welfare and health.

Development of actions flowing from the Global Digital Compact will also need to be set in the context of other global instruments that affect migrants, refugees and displaced persons—in particular, the Global Compact on Refugees^11^ and the Global Compact for Safe, Orderly and Regular Migration.^348^ It will be important to avoid the historical tendency^24^ to create policy sector silos and ensure that policy-making related to digital aspects of migrants, refuges and health is coherent with human rights and humanitarian aims, as well as the SDG principles. Similar considerations of policy coherence and the avoidance of structural barriers apply at all global-to-national levels.^124^^,^^349^ In the European context, implications include the need for coherent policy and action among the major agencies, including the European Union and WHO Regional Office for Europe, and the integration of migrant and refugee data in health information systems in Europe.^55^ The EU's Pact on Migration and Asylum, agreed in May 2024, is a set of new rules for managing migration and establishing a common asylum system at EU level. It includes the Eurodac asylum and migration database. The Eurodac Regulation^350^ turns the existing database into a fully-fledged asylum and migration database, “ensuring clear identification of everyone who enters the EU as an asylum seeker or an irregular migrant”.^351^ The 2024 Regulation also sets the conditions under which requests for the comparison of biometric or alphanumeric data with Eurodac data for the purpose of preventing, detecting, or investigating terrorist offences or other serious criminal offences should be allowed.

The Migration Policy Institute Europe^352^ has emphasised the need for policymakers, civil servants, and others involved in digitalisation efforts to judiciously steer their adoption of the Pact, including by developing appropriate governance frameworks to regulate aspects such as data protection, oversight, and accountability, and creating a strategic vision for the use of new technologies in migration and asylum systems. Eurodac is an EU-wide information system that primarily processes the fingerprints of asylum seekers and irregular migrants apprehended in connection with their irregular border crossing and irregular staying. Its aim is to track secondary movement in the EU by obliging Member States to collect the fingerprints of every asylum seeker over the age of 14 when they apply for international protection. Eurodac fingerprinting does not determine the identity of a person per se, though it does contribute to their identification. Over time, the purposes for which Eurodac is used have been expanded and more categories of personal data added, including facial image, lowering the fingerprinting age to six years, increase in the retention of irregular border crossers’ data from 18 months to 5 years and possibility of transfers of Eurodac data for return purposes.^353^ Critical analysis^354^ has demonstrated that Eurodac is progressively being transformed from an information system of limited aims and capacities into a support tool for a range of EU policies on asylum, resettlement and irregular migration. Use of blockchain can help to strengthen the security and confidentiality of data held and limit access to those authorised, although access may be extended to others (nodes) officially mandated as a result of policy changes. Challenges, where appropriate, need to be made through political, legal, and human rights channels. In an alternative vision for digital technologies that support migrant and refugee health, it has been proposed^242^ that, with regard to forced migration, BC technology could support creation of a global data space in situations of humanitarian catastrophe in an emergency context. Anchored in the security, privacy, and medico-legal regulation of medical data, this could improve communication, overcome gaps in medical data sharing, and empower inter-organisational services or workflows anywhere in the world.

It is evident that addressing the health of migrants and refugees through digital technologies and services needs to be framed in a multi-dimensional context that includes considerations of (1) the entire digital space as it operates globally-to-locally and the extent to which this is accessible and affords opportunities and risks to the users; (2) the rights of migrants and refugees; (3) the health systems and services with which they are able to engage; and (4) personal factors and circumstances of each person. All of these factors are dynamic, evolving and interacting in parallel. Thus, action aiming to increase the benefits and decrease the risks of digital health approaches for migrants and refugees needs to take a comprehensive systems approach, in which the net outcome for the individual emerges as a property of the whole system and cannot be identified only by considering one aspect (e.g., a health need or a tailored digital app) in isolation from others (e.g., security, finances, social or cultural factors). Such a multi-dimensional approach can also help to ensure that the use of digital technologies and services for the health of migrants and refugees is aligned with the overall aim of the SDGs to “leave no-one behind” and specific goals including SDGs 3 (good health and wellbeing), 5 (gender equality), 10 (reduced inequalities), 16 (peace, justice and strong institutions), and 17 (partnerships for the goals).

To summarise key points in this article and provide the basis for advancing the effective, safe and equitable use of digital solutions for migrant and refugee health, we present in Table 1, a number of commendations aimed at the spectrum of actors involved.

**Table 1:** Recommendations regarding digital solutions for migrant and refugee health.

Addressed to	Recommendations
Users/research subjects/participants and their representatives	**Active participation** Individuals and representatives of groups and communities with personal experience of migrating and asylum-seeking should actively engage with those developing and implementing policy, technologies, and services in the public, private and not-for-profit sectors concerned with digital solutions for their health, in order to: • From their experiences of the realities of migration, highlight the ways that digital rights and health rights, both of which are aspects of defined human rights, are not being respected in current practice relating to digital technologies and their applications to migrants and refugees, and apply their knowledge and experience to encourage and facilitate improvement. • Encourage efforts to make digital approaches for their health ‘safe and beneficial by design and in operation’. • Participate directly and actively in all stages of the development, roll-out and evaluation of digital solutions for migrant and refugee health.
Policy makers	**Rights and equity as paramount principles** Having adopted global, regional and national instruments respecting human rights and equity, governments should avoid inconsistency in the application of the principles these instruments contain, especially in the case of migrants and refugees, who are often ‘exceptionalised’ despite commitments to ‘leave no-one behind’. Taking rights and equity as paramount principles in promoting and enabling digital solutions for migrant and refugee health requires: • Ending practices that dehumanise migrants and refugees in all aspects of their treatment, including in the control of borders and in the denial of services that are the basis of fundamental human rights. • Encouraging efforts, through policies, programmes and investments, to make digital approaches for their health ‘safe and beneficial by design and in operation’. • Paying particular attention to instituting regulatory mechanisms to ensure the security, confidentiality and privacy of digital data, which is necessary for the benefit of the whole population, including migrants.
Technology developers/providers/operators	**Accessibility** The device, app, platform or digital intervention should be designed to be as accessible as possible, being mindful of restrictions the potential user may face in access to the hardware, software and connectivity and barriers that may occur due to factors such as language, cultural differences and social situations. **Data security** Design of the device, app, platform or digital intervention should try to minimise if not eliminate exposure of the user to potential identity or surveillance risks. Where possible, it should create a firewall protecting the app or platform from misappropriation of data on the user. Careful consideration should be given to: • What identifying information will be collected and whether it is possible to collect less. • What other data is being collected by the app and/or platform in the background, who is collecting it and what will be done with it presently and later. • If there is a data breach, could the information collected expose the participant/patient to identification and/or risk; and, if so, what ways can be used to minimise this. • How and where the data will be stored and de-identified/anonymized, who will have access to it and when the data will be destroyed. • What safeguards are in place to ensure that data shared via the app or platform is not misappropriated or misused by those who currently have access to it and those who may have access in the future if the app or platform is sold or passed on to a third party. • Whether the app or platform is sufficiently secure so that if another person uses or steals the participant/patient's device, they will not have access to the information and it is easy for the participant/patient to delete the app or their interaction with the service or research from their device?
Digital health service providers	I**nformed consent** When involvement of migrants and refugees in digital services for health requires consent, the provider should ensure that the purpose of the service and the process for and nature of the consent are clearly explained to, and understood by, the user, where necessary in the user's own language; that the user does not feel under pressure to give consent; and that risks associated with taking part in the service, are explained and understood, including risks to health and to the privacy and security of the person's data and identity. **Inclusion** The provider should ensure that use of the app, platform or digital service is inclusive of the full diversity of the intended population and will not exclude certain groups of people due to, for example, factors such as age, disability, documentation status, ethnicity, financial circumstances, gender or LGBQTI status.
Humanitarian assistance provider	States, international agencies, nongovernmental organizations and local community groups providing assistance to migrants, refugees, asylum seekers, and displaced persons need to be aware of the risks as well as the benefits. In minimising risks associated with adoption of digital tools and services, they need to pay attention to inclusion factors, access barriers, challenges in ensuring informed consent and risks to privacy, confidentiality and security that may ensue from the participation of individuals in diverse and sometimes very precarious circumstances, as discussed above.
Researchers, research funders, ethics review committees, academic institutions	**Ethics, diversity, inclusion** Those supporting, designing, approving, and conducting research on digital approaches to the health of migrants and refugees should ensure that: • The research applies ethical principles that are fully adapted to the needs, vulnerabilities and diverse situations experienced by the individuals, with detailed consideration being given to the ethical questions related to data protection, informed consent and exclusion and inclusion criteria listed in Panel 2 in this article. • They adopt participatory research that involves migrants and refugees in all possible stages of the research process, assessing the ‘optimal’ level and type of participation that is most likely to ensure benefit to them and leverage their empowerment. The participatory approach selected should ensure that the research design, conduct, analysis and reporting involve meaningful migrant participation at every possible stage; and that priority is given, in deciding the nature of the research processes and the digital tools employed, to ensuring both benefit to the subjects and protection of their privacy and security. • Effort is made to include the full diversity of the intended population, with attention given to factors that may exclude some people and result both in fostering health inequalities and in created skewed data that can perpetuate inequalities and misconceptions. • Concerted effort is directed to developing an ecosystem that will support and sustain participatory, interdisciplinary, transdisciplinary, and inter-sectoral projects.

## Contributors

SAM and JH conceptualised the manuscript. SAM, JH, and KNM wrote the original draft of the manuscript. All authors contributed to the identification of references, development of the text and reviewed and edited the manuscript. SAM finalised the manuscript and conceptualised and constructed the Figure.

## Declaration of interests

We declare no competing interests.
